# Respecting, protecting and fulfilling the human right to health

**DOI:** 10.1186/s12939-022-01634-3

**Published:** 2022-03-15

**Authors:** Zahara Nampewo, Jennifer Heaven Mike, Jonathan Wolff

**Affiliations:** Makerere University Faculty of Law: Makerere University School of Law, Kampala, Uganda

**Keywords:** Human right to health, Respect, Protect, Fulfil, Implementation

## Abstract

**Background:**

Human rights are best protected, promoted and guaranteed when they can compel binding and enforceability duty. One prominent criticism of category of human rights which includes the human right to health is that it is difficult, to assign the duties that correspond to these rights, because of stark disparity in how the main duty bearers approach their duties.

**Methods:**

This paper adopts a doctrinal approach to examine and evaluate the duties to the right to health. The method in this study entails a detailed literature search to systematically evaluate the legal implications, regulations, arguments and policy regarding the nature of the obligation to the right to health. This study also engages with normative and philosophical aspects of human rights.

**Results:**

This paper posits that human rights protect against common, serious, and remediable threats and risks, and ensure that there are remedies from governments and third parties. However, it is difficult to compel duties especially in regard to the right to health. First it is not easy to achieve a uniform standard for duty bearers implied by the words ‘highest attainable physical and mental health.’ Theorists discussed in the paper outline views of what this could mean, from serious to common health concerns. Second, the right to health is not a legally established right in many jurisdictions, making it difficult to enforce. This paper outlines different layers of state and non-state legal duty bearers to enforce the right to health.

**Conclusion:**

The duty to respect, protect, fulfil and even remedy the right to health, will often be meaningless in practice without a clear identification of the necessary duty bearers to enforce them. The law is the starting point for this to not only enshrine this right as a legally enforceable one but also to clearly identify duty bearers. Without this, the human right to health as outlined under international and regional human rights law generates an implausible, or even impossible, profusion of duties. There remains much work still to be done especially on the moral and legal fronts in order to fully guarantee this right.

**Trial Registration:**

Not applicable

Our work does not report results of a health care intervention on human participants. Registration is therefore not applicable.

## Introduction

### Background

Health is both a human right in itself and an essential means for the realisation of other human rights. [1, 2]. Good health is one of the many aspects of human wellbeing that is necessary for the enjoyment of human rights. Health also plays a pivotal role in empowering people to pursue other activities that will enhance their welfare [3, 4]. In this respect, a healthy person is in a better position to practically engage in activities he or she finds useful, improve their living standards, increase their life chances and also enjoy other human rights. As an essential state of wellbeing, health is also a means by which people can undertake social, economic and cultural activities as well partake in civil and political activities, and, as a basic human right, health is an essential, fundamental and indispensable state of wellbeing [4]. The right to health is, therefore, one of the cornerstones for the enhancement and improvement of overall wellbeing and human development.

The guarantees and articulation of human rights to health are acknowledged in several human rights laws and instruments [5]. To give proper meaning to the right to health, some parties are tasked with the duty of safeguarding, protecting, guaranteeing and fulfilling and also, providing remedies for any breach of the rights. These duties accrue when states become parties to international treaties. This was reaffirmed by the Declaration on the Right and Responsibility of Individuals, Groups and Organs of Society to Promote and Protect Universally Recognized Human Rights and Fundamental Freedoms adopted by the United Nations General Assembly in 1998 [6].

The world's understanding of the action needed to advance human rights is deeply structured by the ‘respect, protect, and fulfil’ framework [7–11]. According to Reeves, it entails duties to: ‘(1) *respect* rights, that is, avoid harming, or introducing deprivation of concern to, protected interests, (2) *protect* rights, that is, adequately ensure that others respect rights, and (3) *provide*, that is, aid those whose protected interests are experiencing remediable setbacks’ [12]. Here we will also discuss a fourth type of duty, related to the duty to protect, which is to *implement* through a functioning legal system. Despite the clear delineation, the duty to fulfil the right to health remains an issue across the world which calls into question how the right to health can fulfil its objective. Arguably, there is stark disparity in the ways in which the main duty bearers approach their duties, which reinforces the common argument against economic, social, and cultural rights, including the right to health, that it generates too many, or the wrong type, of duties. Assessing this critique is one of the main aims of this paper.

In the same vein, this paper accepts the view that the human right to health generates a range of obligations on individuals, states, corporations, NGOs and the international community, insofar as each of them have identifiable human rights duties. However, regarding the specific obligations of each party, it is arguable that the state, through its laws and implementation authorities must take the lead in fulfilling the duties necessary to effectively guarantee this right. We present the argument here by looking at both the underlying philosophical questions of the nature of duties, and at how human rights duties are given legal effect, especially in the context of African legal systems.

The paper is divided into four parts. The first part underscores the philosophical and normative argument for establishing human rights and the basis upon which human rights duties are given legal effect,setting the tone for the following arguments in the paper. Within the context of the right to health, the second part interrogates the nature of duties to the right. The third part relies on various jurisprudence to make a case for the implementation and enforcement of the right to health as an imperative obligation of states. The final part concludes with a recommendation for a rightsbased approach to fulfilling the duty to the right to health.

## Methods

The doctrinal methodology in this research is employed to examine the underlying philosophical questions about the nature of the duties and how the obligations to the right to health are given effect, especially in the context of legal systems. We interrogate this by focusing on the human right to health as one of the cornerstones for the enhancement and improvement of overall social, cultural, economic welfare and human development. The doctrinal approach is ‘a detailed and highly technical commentary upon, and systematic exposition of, the context of legal doctrine’ [13]. A doctrinal research or ‘black letter’ research involves a systematic and analytical study of legal rules, judicial decisions and authoritative materials in relation to certain issues raised. This methodology further employs a desk review of existing literature that serve an important function of providing a foundation upon which to build the subsequent arguments that are made in this research. This approach essentially allows the researchers to critically analyse the issues, the meanings and implications of human rights and the obligations which underpin them. The main sources of data for this research are international and national human rights instruments and other statutes that make provisions for human rights, and cases and decisions that touch upon the right to health. This research also considers books, scholarly articles, and any other sources relevant to the issues in this paper.

## Results

### The need for human rights

While looking in detail at mechanisms of implementation of the right to health, it is also worth considering why it is that some moral concerns are regarded as so important that they are elevated to the status of universal human rights, meaning other people, organizations, legal entities etc., then have the assigned duty, sometimes the enforceable assigned duty, to satisfy the requirements of such rights. While there is controversy about the foundations of human rights, it can be agreed that at least one of the main purposes of human rights is to safeguard vulnerable people who are in danger of being neglected or even persecuted unless there is a moral and legal scaffolding to protect their interests. Human rights are needed so that their interests do not recede from view, or their difficulties are not considered merely problems they have brought on themselves. For example, women in prison may be forced to wear handcuffs while giving birth. What is the public response? Some will be horrified, and believe that the human right to dignity is violated, others conclude that the mother herself is to blame as she did something to put her in prison in the first place. The latter opinion tends to be more likely if the woman in question is not a citizen of the country in question, or is a member of a minority. Human rights however force us to take the perspective of the victim. Whatever she has done, she is still entitled to dignity and protection. This can be very uncomfortable for those in power. Indeed, members of governments often are the first to try to undermine human rights discourse, as it is one of the mechanisms used to hold them to account [1]. Even when the abstract idea of human rights is popular, when they are pursued in particular cases they are often derided or parodied. This is all the more reason why they are needed.

In international law the human right to health makes a relatively subdued appearance in the Universal Declaration of Human Rights (UDHR). Humans are declared to have a right to a standard of living adequate for health, and, insightfully, a right to various other underlying determinants of health, such as food, clothing and housing, as well as medical care [14]. However, the drafters of the later International Covenant on Economic, Social and Cultural Rights (ICESCR) went much further, declaring a universal right to the highest attainable standard of physical and mental health [15]. This statement however is a challenge for defenders of the human right to health, foras Onora O’Neill has argued: “*what is to be made of the idea of the ‘highest attainable standard of health’? Consider a low resource environment such as rural India or sub-Saharan Africa. If we mean the globally highest attainable standard, then we are setting a utopian standard. If we mean the locally highest attainable standard are we not setting our target far too low?”* [16]

A related difficulty is pointed out by Joseph Raz, although overall he is much more sympathetic to the human right to health than O’Neill. He points out that the notion of the ‘highest attainable’ standard does not specify whether it is the ‘highest attainable’ or ‘highest attainable, given proper weight to all other considerations, including other moral rights and worth-while goals’ [17].

Clearly, much work is needed to clearly chart a course for human rights. One way forward may be to accept Henry Shue’s notion that human rights protect against common, serious, and remediable threats [11] which, in this case, are common, serious, and remediable threats to health. To take them in reverse order, inclusion of *remediable* threats is obvious at least in terms of the duties it generates; if nothing can be done, and it is not the sort of harm for which compensation is possible, then it doesn’t generate any clear possible duty, apart, perhaps from the duty to research how to meet similar threats in future. It may be argued, however, that a human rights violation may still exist even if nothing can be done. There is a good case for accepting this, as it provides a way of keeping up pressure to look for a remedy. Remediable, therefore, should be understood as remediable or compensable in principle, in part or whole, even if nothing can be done at the moment.

The inclusion of the idea of *serious* threats, again, is obvious, in order to leave out trivial threats. There may however be some ambiguity here. Does trivial mean low-probability, or low-harm? Some combination of these is likely the best way forward, although how in practice the line is drawn will be a matter of contention. The inclusion of *common* threats, however, may seem more debatable. Why common? Why not a human right against unusual threats? Arguably the notion of the idea of a threat being common brings out the underlying egalitarianism of human rights doctrine. In most societies members of the elite rarely need to appeal to their human rights, except in cases of deliberate political persecution, if, for example, they are the member of an opposition party. The elite are normally able to protect their own interests again common threats. There will however be groups in society who need assistance to achieve even a basic level of protection, and human rights are designed to support those in such vulnerable positions. Human rights protect against mundane, ordinary risks, not exotic one-off harms [18].

Despite varying interpretations of these threats, the key notion is that human rights are protections especially for the vulnerable. We resist saying protections for minorities, for there are times when majorities can lack power, and need the protection of human rights. The idea that human rights offer special protection for the vulnerable may however seem to be in tension with the idea that human rights are universal. But, as discussed above, it is precisely because they are universal that human rights can protect minorities. The universality of human rights is a counter to the pattern that can easily be fallen into, when there is, de facto, one law for the rich or powerful and another for the poor or vulnerable. For example, the police and security services may take steps to protect the elite from a common threat of serious theft or assault, but leave the poor to fend for themselves. If there are steps that could be taken to improve the situation, i.e. the threat is, to some degree, remediable, and the threat is serious and common, if only the privileged are protected. this is where human rights claims take root. The vulnerable are ignored, or, even worse, suffer deliberate discrimination, and thereby their rights are violated. The vulnerable do not have access to what should be universal and is enjoyed by the rich. This concept remains true, however large or small the unprotected group is, provided that there is a protected elite.

Within the context of health, the threat of tuberculosis, for example, is common in many countries [19]. It is serious, and remediable in most cases through prevention or treatment. In many countries those who are rich, or belong to the ruling families, or work for the government, are protected. First, they live in conditions that are less conducive to the spread of infection, and second, have access to high quality treatment. Those outside these charmed circles are far more likely to fall ill, and far less likely to receive appropriate treatment, and thereby have a case that their human right to health has been ignored or violated. Hence, they are not protected from a common, serious, and remediable threat, while others are.

If one compares this with the case of a very rare disease, of equal virulence, that can strike rich or poor, if the rich are treated and the poor are not, there is an argument that the human right to health of the poor is violated. If however, no one is treated, there is much less strength to the argument that there has been a human rights violation. Rather, something very unfortunate and troubling has happened but as there is no pattern as to whom has been neglected it is harder to argue that human rights have been violated.

In this example, the requirement that the threat is common is an imperfect but useful proxy for another idea—that being especially vulnerable to the threat is a consequence of being a member of a vulnerable group. Here human rights should be a protection against this form of double vulnerability. Members of vulnerable groups may well suffer additional risks or threats that the non-vulnerable do not. This scenario is common in health care and has been illustrated by the COVID-19 pandemic where those in the lower socioeconomic strata had greater comorbidities at least in part because of structural inequities, which increased their susceptibility to infection and severe disease [20]. This is why human rights are needed. If this is correct, it explains why claims from the wealthy that their human rights have been violated so often ring hollow (again outside cases of deliberate political discrimination). In comparison to whom have the wealthy been treated badly? There may be an answer, but it needs spelling out. This also explains why, even though many people claim to be in favour of human rights, they tend to be much less in favour of those people who pursue human rights claims, because those claims are pursued, most likely, by those who are unpopular; refugees, outcasts, prisoners, members of minorities, etc. [21].

### Human rights duty holders

A common argument against economic, social, and cultural rights, which includes the human right to health, is that they generate too many, or the wrong type, of duties. The right to health seems to be a positive right to assistance, and if human rights are universal then it seems they create universal positive obligations. Does this mean that any individual, is a human rights violator if they don’t keep everyone in the world alive and in good health? The same would apply for every other economic, social, or cultural right. Such an overwhelming proliferation of duties seems intolerable, and has been used as a *reductio ad absurdum* (Latin: “reduction to absurdity”) of economic, social, and cultural rights.

In the current discussion, however, this argument is rightly regarded as a cheap shot, as it fails to differentiate the different types of duties that can be associated with a right. Regarding the right to health, General Comment 14: The Right to the Highest Attainable Standard of Health (Art. 12 of the Covenant) expatiates that the right to health, like other rights, generates a tri-partite structure of duties: to respect, protect and fulfil [1]. It can be argued that the source of this account of duties is, in fact, Henry Shue’s book Basic Rights [11] as modified by others [22]. As a way of overcoming what Shue regarded as a misleading and simplistic division of rights into ‘negative’ requiring duties of non-intervention, and ‘positive’ requiring duties of active assistance, Shue pointed out that the rights he was interested in – to liberty, security and subsistence – generate duties to ‘avoid’ certain types of behaviour, to ‘protect’ individuals from violations by others, and to ‘aid’ some individuals in achieving their rights [11]. These duties do not have to be held by the same party, although often they will be. Even ‘negative’ rights, such as the right to security, require positive action by governments, such as the provision of a police force.

What, then does this mean for the right to health, and in particular, the nature of the duty holder? One way of shortcutting the ‘proliferation of duties’ objection is the suggestion that in the first instance the duty holder in relation to the human right to health is the state, and if the state is unable to deliver, the duty then falls on the international community [23]. This suggestion could be criticised, perhaps, as an over-generalisation. Apart from the problem of people who are stateless, which can be answered by this approach, it has been suggested that multi-national companies as well as ordinary citizens can also have human rights duties [4].

Indeed, non-state duty bearers can be divided into the following groups:

1. Primary legal and care duty-bearers – e.g. parents for children, teachers for students, police for crime suspects, doctors/nurses for patients, employers for employees;
2. Secondary duty-bearers – e.g. institutions and organizations with immediate jurisdiction over the primary duty-bearers e.g. school principals, community organizations, hospital administrations, etc.;
3. Tertiary duty-bearers – e.g. institutions and organizations at a higher level / with more remote jurisdiction (NGOs, aid agencies, private sector organizations);
4. External duty-bearers – e.g. countries, institutions, organizations with no direct involvement e.g. WTO, UN, NGOs, Security Council, EU, African Union etc.
5. Private individuals, corporations, business entities.

The international community has yet to succinctly outline the nature of the duties that all the above parties will bear towards the right to health. The focus has mainly been on the duty of states. In the same vein, not many academic discussions have focused on how the aforementioned persons and entities can play a role in guaranteeing the right to health, and in particular the duties associated with the rights. Because states sign treaties, they are ultimately the legal duty holders (unless a law specifically identifies any of these non-state actor as having legal duty), however, one cannot discount the role of non-state actors in promoting, protecting and respecting the right to health, as exemplified by the debate on the commercial determinants of health [24].

Buttressing the categorisation of duty bearers as mentioned above, General Comment No. 14: The Right to the Highest Attainable Standard of Health (Art. 12) [1] in Para 42 stipulates that although States parties are ultimately accountable for compliance with human rights, all members of society—individuals, including health professionals, families, local communities, intergovernmental and non-governmental organizations, civil society organizations, as well as the private business sector—have responsibilities regarding the realization of the right to health. With regards to the core obligations, the Committee para 45 emphasized that ‘[…] it is particularly incumbent on States parties and other actors in a position to assist, to provide “international assistance and cooperation, especially economic and technical” which enable developing countries to fulfil their core and other obligations.’ Noticeably, the Comment does not expound on the nature of the non-state entities’ obligation in the exact terms as that of states. States are left with the discretion on how these non-state entities discharge their human right obligations. The Comment however, states that the State should provide an environment which facilitates the discharge of their responsibilities (Para 42).

The human rights responsibility of international assistance and cooperation in health is also gaining significant attention, and has become more urgent during the COVID 19 pandemic. This has often been analysed through the lens of high- and low- income states, and multilateral and bilateral trade agreements [25]. The General Comment No 14 on the Right to Health cited above [1] lays the foundation for an international commitment to the right to health, outside one’s own state. On the basis of their international obligations in relation to human rights, States are enjoined to respect the enjoyment of the right to health in other countries (Para 39). The Committee also clarified that states have a duty to prevent the violation of the right by third parties in other countries if they are able to influence these third parties through legal and political means, in accordance with the Charter of the United Nations and applicable international law (Para 39). Specifically, states should facilitate access to essential health facilities, goods and services in other countries, wherever possible, and provide the necessary aid when required’ within the context of available resources at their disposal.

Of particular note are the duties of corporations, businesses and third parties. Do third persons or non-state actors and corporations have a moral, or even a legally binding, duty in respect of the right to health? It has been suggested that they are obligated to respect and contribute to promoting human rights [26]. Accordingly, within the scope of their business operations, business enterprises and corporations, and third party service providers should respect, protect, fulfil and support the human rights of everyone [21]. Multi-national companies are often accused of being human rights violators, for example through be exploitative or dangerous work conditions, severe pollution, complicity in theft, corruption, or money laundering [27].

In this manner, the UN Norms for corporations recognized the responsibilities of corporations and business enterprises to respect, promote and secure human rights. The UN Norms for Corporations and Businesses further indicates that states have a general duty to ensure that corporations and business enterprises respect and promote human rights [28]. Likewise, state parties are required to prevent violations of human rights, including the right to health, by third parties, organisations and enterprises operating within the rights granted by states [10]. The issue remains, how corporations and third parties be made to contribute to the human right to health? Another question in this respect is whether and how states, as principal duty bearers, can legally compel third parties or corporations to protect and promote human right. Moreover, what kind of duty(s) are they expected to undertake? Specifically, can they protect, respect and guarantee the right to health? If so, is the standard same as that of States? On a smaller scale, ordinary individuals are sometimes accused of human rights abuses. Although not all abuse of rights is abuse of human rights, it is all too easy to slip from ordinary cases of rights abuse to human rights abuse. It seems plausible therefore that multi-nationals and citizens can have human rights duties. Does this, then, put them on a par with states in terms of their duties? And does this return us to the problem of proliferation?

Here the distinction between duties to respect, protect, and fulfil becomes relevant. It can be conceded that entities beyond the state can have duties to respect the human right to health. This essentially means not engaging in behaviour that threatens the health of others. Although it may seem an absurd to claim that an individual’s badly polluting car fails to respect another’s right to health, a whole fleet of badly polluting taxi cabs dominating a neighbourhood, begins to enter the realm of potential human rights claims. Non-state actors can therefore non-problematically be considered to have duties to *respect* the human right to health.

The claims that non-state actors have such duties to *protect* and *fulfil* the right to health however are less clear and may be less direct. The duty to protect is, in the first instance, a duty to create effective institutions to provide a reasonable guarantee of health. The duty to protect, generally, in the first instance is a call to put effective institutions in place. This would be expected this to fall on the government, although as a stop-gap international organisations such as the United Nations or the WHO can be required to step in. Organisations act through individuals who are officials within those organisations, and there are corporations and individuals who are influential within their community who can input into this right. Individuals can therefore protect the right to health bypromoting access to health care and health related services provided by third parties, refusing harmful social or traditional practices such as female genital mutilation, and enhancing information on health. This may also include the role of individuals and corporations to challenge legislation that does not promote the human right to health, including discriminatory legislation.

Indeed, the African Charter on Human and Peoples Rights (African Charter) envisages the responsibilities of individuals in achieving human rights in Chapter II [29]. Accordingly, Articles 27, 28 and 29 exhort a duty on individuals towards their families, society, state and communities. This duty includes the responsibility to respect the rights of others, preserve and strengthen social and national solidarity. Additionally, the African Children’s Charter under Article 31 imposes a range of duties on children [30]. Thus individuals can, through their concerted actions, promote the right to health of others. In sum, individuals and corporations can have human rights duties to protect, but those duties will often be different in content to those of the state and international organizations.

Finally, the duty to *fulfil* often requires the provision of services. This duty often gives rise to the proliferation objection [31]. If every individual has a human right to health, then there will be obligations to fulfil that right in terms of provision of directed services. But on whom do those duties fall? If a person in failing health can call on every other individual for direct assistance, then the proliferation objection hits hard. It is too demanding to make individuals responsible for providing whatever it takes to attempt to bring everyone else back to health. But rather than reject the human right to health on this basis, this requires understanding of the division of labour in human rights duties. As discussed above, it does not seem problematic to say that individuals have a duty to respect the human right to health, which means not engaging in action that creates serious health threats to others, and even to assist in protection of the health of others, in some cases. But the duty to fulfil seems to need concerted, sustained action, and the default position is that these duties fall on the state.

Breakey [32] introduces another challenge with regard to duties as outlined in General Comment 14 regarding realizing legal accountability for those to whom duties are assigned. Legal accountability means that an authority may compel compliance with the duty, or punish those who do not perform it [33]. Rights theories prize legal accountability for two reasons. First, making duty-bearers legally accountable for failing in their duties increases the sense in which rights can be guaranteed to the right-holders – that is, the likelihood that the right will be fulfilled, and for right-holders to be able to rely on this fact. However, this does not come with any guarantees. Second, legal accountability authorises an (arguably) appropriate retributive response to the profound moral wrongfulness of violating others' rights. At best, however, these considerations only require that some of the duties based on the right must possess legal accountability. With respect to guarantees, coercive avenues may be just one part of a range of strategies employed to guarantee rights. Legal accountability, at best, merely expresses a state's commitment to produce a result, which is not at all the same thing as producing that result [32].

It is also important to note that the obligations of states under the directive of international assistance are not easy to achieve. For instance, whist the UN Charter [34] in Article 1(3) talks of international co-operation in solving international problems of an economic, social, cultural, or humanitarian character, this is wishful thinking and is hard to enforce. When states fail to take part in joint action in co-operation with the Organization for the achievement of human rights they can barely be brought to task in accounting for this which further complicates the discussion on duties [35].

### Duty of the state to the right to health

Most scholars and case decisions agree that the duties of the right to health sit squarely on the shoulders of the state who should ensure the fair provision of the facilities, services and products necessary to promote and safeguard the right to health, through minimum core obligations [36]. In *Purohit and Another v The Gambia* [37] the African Commission held that The Gambia fell short of satisfying the requirements of Articles 16 and 18(4) of the African Charter in guaranteeing the enjoyment of the right to health which is crucial to the realisation of other fundamental rights and freedoms. The African Charter has also played an important role in imposing a human rights responsibility on the Nigerian government to respect the right to health and provide medical care to its citizens. In *Media Rights Agenda and Others v Nigeria* [38], the Commission took the view that the denial of an incarcerated suspect’s access to medical care while his health was deteriorating is a clear violation of the right to health under Article 16 of the Charter.

The Costa Rican Supreme Court in *Mr William García Álvarez v Caja Costarricense de Seguro* [39] also ruled in favour of the plaintiff, an HIV-positive person who was refused antiretroviral treatment by the social security institution. The plaintiffs argued that the treatments were expensive in the private sector and so refusal to provide them by the institution and inaccessibility was a violation of the right to life and health [39]. The judge, in the ruling in favour of the plaintiff, decided that:

If the right to life is especially protected in each modern State and with the right to health, any economic criteria that pretends to deny the exercise of those rights, has to be of second importance […] without right to life, all the remaining rights would be useless [39].

The jurisprudence of South Africa’s Constitutional court has broken new grounds on the obligation of the state to the right to health. In the South African cases of *Treatment Action Campaign and others v Minister of Health and others* and subsequent appeal to the Constitutional Court (*Minister of Health and others v Treatment Action Campaign and others)* [13, 40], the court took the time to consider the legal obligation of the state to enforce socio-economic rights and stressed that the state is under a constitutional duty to take all necessary and reasonable actions to comply with the provision of the right to health.

The Indian Supreme Court has also made notable pronouncement and paved the way for the enforcement of the right on the right to health. In *Samity v State of Bengal* [41], for instance, access to timely healthcare necessary to preserve life was upheld by the Indian Supreme Court. Deciding on the basis of the right to life, the court held that the right includes an obligation to provide access to medical treatments to preserve human life as a ‘constitutional obligation of the state to provide adequate medical services to the people.’ (Paragraphs 9, 15–16). Notably, the court held that this duty on the state is irrespective of financial and resource constraints. The Supreme Court stated this as follows:

It is no doubt true that financial resources are needed for providing these facilities. But at the same time it cannot be ignored that it is the constitutional obligation of the State to provide adequate medical services to the people. Whatever is necessary for this purpose has to be done. In the context of the constitutional obligation to provide free legal aid to a poor accused this Court has held that the State cannot avoid its constitutional obligation in that regard on account of financial constraints. (Para 16).

Similarly, in *Poltoratskiy v Ukraine* [42] the European Commission of Human Rights (ECHR) also took the view that ‘lack of resources cannot in principle justify prison conditions which are so poor as to reach the threshold of treatment contrary to Article 3 of the Convention.’ (Para 148).

Despite the judicial affirmation of a duty to the right to health, one question however, remains unanswered: What happens then if the state is unable to act? Within human rights law the notion of ‘progressive realisation’ is established, allowing states to act within their resources, making progressive, concrete steps to achieve full realisation [43]. In many cases they may call on the international community for assistance, but one can also look in the other direction at individuals and corporations within the state. Where a state lacks financial resources it can perhaps draw on other resources that can only be offered by corporations and individuals, such as skills, commitment, education and so on, in order to ensure that the right to health is as fully realized as it can be without discrimination of any kind. The notion of progressive realization within available resources must not be viewed as an excuse to defeat or deny economic, social and cultural rights including the right to health, but as an opportunity to expand the scope of duty bearers in order to ensure maximum realization of the right to health.

Furthermore, the duty to protect human rights, it can be argued [44, 45] entails the responsibility to avoid and mitigate any adverse human rights impact that non-state entities such as pharmaceutical companies and their business activities may cause or contribute to, and is linked to their operations, products or services [26]. Positively, pharmaceutical companies can support the state to fulfil, respect and protect the right to health by providing the means for the realisation of the right to health [46]. Through their pharmaceutical R&D and production undertakings, the drugs they produce can facilitate the availability of drugs for the realisation of the right to health. It is further argued, however, that their contribution to the human right to health goes beyond providing the facilities and goods (medicines) necessary for the enjoyment of this right. This responsibility extends to refraining from any act or policy that will obstruct access to affordable and available medicines, given that their business and marketing practices could limit access to medicines.

The UN’s Interpretive Guide on ‘The corporate responsibility to respect human rights’ emphasises in this regard that ‘[f]or pharmaceutical companies, the right to health will be particularly salient’ [47]. However, whether they actually owe this responsibility as an enforceable legal duty or mere corporate social responsibility, and how to measure the responsibility of non-state entities and individual companies in this regard may vary considerably, depending on where they operate and whether national laws impose this duty on them. Identifying the duties and role of non-state actions to the right to health is one thing, ensuring that they actually play this role is another. The state, as the primary duty bearer, can put into place domestic measures and legislation to compel such a duty. It however, waits to be seen how this will play out in practice [48].

Even where a country is not resource constrained, there are cases where corporations arguably have obligations to fulfil human rights to health duties. For example, if a university or pharmaceutical company is conducting a large-scale trial, they may then acquire health-related obligations that go beyond the strict confines of their research (for example if they incidentally discover non-related health conditions of participants in their trials [49].

Digging deeper, and trying to avoid sweeping generalisations, there can be specific obligations to fulfil the human right to health that can also fall on individuals and corporations. For instance, parents have a duty to make their children available for vaccinations, which is a human right for children [50]. Without these vaccinations, perhaps the children would suffer later violations of their right to health. In another example, in many countries in Sub Saharan Africa, if the respect for and actual duty regarding sexual and reproductive health is not supported by individuals at family and community levels, there is little that the state can do [51]. This calls for duties beyond the state party. Furthermore, whereas physical health might point towards duties mostly on the state, individuals have a central role to play in ensuring the right to mental health at family and community level. This is a critically important issue, but often overlooked. We still lack the know-how to impact on mental health at scale, and the best place for action is within the family and community.

Finally, even if there is no specific action called upon individuals, citizens are corporate members of the state, and so the state’s responsibility is the citizen’s responsibility. How citizens fulfil their responsibilities is different from the state. Citizens must pay taxes, vote for, or otherwise support, governments that take human rights duties seriously. Citizens must remain vigilant about whether those duties are being followed. The point is that, instead of stumbling over the proliferation of human right to health duties, the distinction between the obligations to respect, protect, and fulfil, and the different roles different individuals and organisations can have in relation to those duties must be understood. Of course, disagreements, disputes and grey areas remain, but these must be recognized and tackled.

### Human rights legal implementation

The more concrete issue of how the right to health is implemented and enforced also requires discussion. While many municipal legal systems recognize the right to health explicitly or tacitly, the statutory enforcement of the right to health varies across counties. A cursory survey of the legal provisions indicates that many countries make explicit reference to the right without sharing the value-system of the international community [52]. As the WHO has observed, the right to health in a constitution that focuses on the duties of the citizens is not the same as the right to health in a traditional liberal constitution where the focus is on the rights of the citizen. [53] In many national contexts, the disparity between the state’s obligations to civil and political rights and socio-economic and cultural rights is in favour of political rights. The ethos or spirit of constitutions may indicate an intention to safeguard the health of the people but the enforcement mechanism vary on political grounds, with emphasis laid on civil and political rights. Countries such as Botswana, Cameroon, Djibouti, Lesotho and Costa Rica still favour the traditional approach that recognizes civil and political rights as fundamental rights and relegate the economic, social and cultural rights in their constitutions [54].

In some countries, particularly the sub-Saharan African Region, the right to health, even where it is mentioned in the constitution, is an unenforceable right against the State. African countries such as Malawi, Tanzania, Namibia and Nigeria recognise socio-economic and cultural rights as mere fundamental objectives and directive principles of state policy to be implemented progressively. The constitutions of Comoros, Mauritania, Cameroon and several other Francophone African Countries make a broad commitment to human rights in the preamble without specifically entrenching them in the Bill of rights [55]. In Nigeria for example, although the necessity of directing state policies towards facilitating access to medical and healthcare facilities to further the material wellbeing of Nigerians is mentioned in the Constitution, the status of this provision as a non-justiciable entitlement robs the provisions of a judicial recourse to compel government compliance, action and enforcement. Many authors have argued in this regard that there is no right to health in the Constitution of the country [56, 57]. Furthermore, the core international human rights instruments that make succinct provision for the right to health has not been domesticated into the corpus of laws in the country. This is not to say there is no right to health in Nigeria, as the African Charter which makes provisions for the right to health forms a part of the laws of the country, as upheld by African Commission on Human and People’s Rights judicial [58]. However, because the Constitution simply states that the provisions on health are Fundamental Objectives and Directive Principles, which the Nigerian state aims to achieve for all its citizens, the Constitution is unable to compel a binding duty on the government to safeguard and guarantee the rights of its citizens. As mentioned earlier, human rights carry with them obligations for the state to respect, protect, fulfil and furthermore to implement. This duty includes ensuring that third parties, policies and laws do not interfere with the enjoyment of the right. As justiciable human rights, the provisions on health would also avail Nigerians with the opportunity and legal recourse to measure the performance of the government, authorities and third parties who infringe on those rights. However, the failure to guarantee the provisions on health as human rights under the Constitution means that the provisions may not make a full meaningful impact on all Nigerians, especially since the provisions are non-justiciable. This is also the case in countries that do not make the right to health a constitutional justiciable right.

In the same vein, there is no right to health, per se, in the Indian constitution. Indeed, the Fundamental Objectives and Directives in Chapter II of the Nigerian Constitution (including the provision on health and medical care) is borrowed from the Indian Constitution (1948) and was first included in the 1979 Nigerian Constitution. Nonetheless, the Supreme Court of India has interpreted the Constitution’s article on the protection of life and personal liberty to include access to health care into the article’s scope [59]. In the aforementioned case of *Samity v State of Bengal*, for instance, the court proactively ruled that access to timely healthcare is an imperative means to preserving life and pursuing other human development objectives [60]. In that case, Samity fell off a train and suffered serious head injuries. The necessary health facilities (including vacant bed) to treat him were not available in six hospitals. The Court held that the failure on the part of the government to provide timely medical care to a person in need of such treatment results in a violation of his right to life guaranteed in Article 21 of the Indian Constitution, citing a ‘constitutional obligation of the state to provide adequate medical services to the people.’ This progressive judicial interpretation of health as an intrinsic aspect of human rights is worth emulation in countries that do not explicitly recognize the right to health in their constitutions and laws. In the Sri Lankan Constitution, health care is included only as an obligation of the provincial councils, and not as a right [53]. However, health care as well as education are free in principle and contribute to consistently improving indicators in these areas. The constitutions of Bhutan, Bangladesh, and Myanmar do not recognize the right to health as a fundamental right, nevertheless, compel the state to provide health services or in some cases, more indirectly to improve public health. Importantly, although the right to health may not been included as a positive right in some constitutions, other national legislation guaranteeing this right might be in place, or access to healthcare may be treated de facto as a right.

Thus as it stands, the provision on healthcare in some countries is a mere political objective and goal, devoid of a concrete redress mechanism against the duty bearers to guarantee the enjoyment of these important provisions on health in countries that do not attach legal enforcement mechanisms to them. This leads academicians and legal specialists to criticize the categorization of the obligations to which the provision on healthcare in the non-enforceable parts of many Constitutions as a ‘toothless bulldog’ that barks but cannot bite because there are no concrete enforcement mechanisms attached to the health objective [56]. Legal guarantees and corresponding legal enforcement mechanisms are central to the right to health, as with other human rights. Human Rights are best enjoyed where they are secured. Moreover, a claim can only become a right if vested with prior recognition by law; otherwise it cannot be legitimately enforced as a right. Devoid of legal enforcement, healthcare provisions may be equated to mere exhortative appeals or abstract rights, lacking compelling enforcement or even sanctions upon breach.

Ultimately, however, the constitutions are the supreme laws of countries. In the case of any contradiction or inconsistency between any other national laws and provisions of the constitution, the stipulation in the constitution prevails. It is therefore imperative that the right to health’ or ‘right to healthcare’ is specifically mentioned as a justiciable fundamental right in national constitutions to compel a binding duty and guarantee the obligations and duties to respect, protect and fulfil human rights.

## Discussion

For several reasons, the inclusion of the right to health in national constitutions is central to the type of duties accompanying the right and the means of enforcing them. A rights-based approach to health signals a paradigm shift to using human rights as pervasive human rhetoric to mandate effective actions by the state and other duty bearers. When citizens become aware of human rights as a pervasive value of a democratic society and assume their role as rights holders, they will take actions to hold the states accountable to improve health service delivery. Human Rights Impacts Assessments (HRIAS) can be used to assess the direct and indirect impacts of government and its authorities’ actions which affect the right to health. Government ministers and officials may be reluctant to be held accountable by a ‘right to health’ obligation because human rights would then hold the government to account.

The courts can adopt a rights-based approach to interpret and enforce matters bordering on the rights to health, life and the dignity of the human person. In recent times, a number of national court decisions in have provided clear reference points on the state’s obligation to the right to health, within the context of the state’s obligation to respect and promote human rights. In the aforementioned cases of *South African cases of Treatment Action Campaign and others v Minister of Health and others* and *Minister of Health and others v Treatment Action Campaign and others* [13, 40] the court decided in favour of the plaintiffs and stated that the restriction which affected the availability and accessibility of essential medicines for women and children violates the Constitutional human right provisions and constituted an ‘unjustifiable barrier to progressive realization of the right to health care.’ In this regard, the court decided that, while it is practically impossible to give everyone access to a ‘care service immediately’ (according to the minimum core obligation), the state is under a duty to reasonably provide access to socio-economic rights on a progressive basis. Although the delineation of this reasonable standard was not clearly defined by the court, it stated that that government is required to undertake all reasonable measures to eliminate or reduce the condition and ‘large areas of severe deprivation that afflict our society.’ Notably, the Court relied on international treaties (ICECSR) to interpret the state’s obligation to adopt ‘reasonable measures’ to implement the right to health. The court was able to adopt this progressive interpretation of the right because the right to health is recognized as a constitutional right in South Africa. Thus if the right to health is incorporated in the constitution as a fundamental right (positive right), it can be enforced in a court of law, rather than leaving it as a hortatory health development objective of the state.

Gains have also been made in linking the right to health with other rights, most especially the right to life. In the Kenyan case of *Patricia Asero and Ors v AG* [42], the court declared that the rights to life, dignity and health are “inextricably bound” and that without health, the right to life would be in jeopardy. In this case, the Petitioners were citizens of Kenya living with HIV. They claimed that the anti-counterfeiting legislation restricted their access to affordable, essential medicines, including generic medicines for HIV and AIDS, and therefore violated their fundamental rights to life, dignity and health under the constitution of Kenya. The Court noted that General Comment 14 affirmed the right to health as embracing a wide range of socio-economic factors that promote conditions in which people can lead a healthy life.” Additionally, in *Centre for Health Human Rights & Development and 3 Ors v Attorney General* [61] the petitioners challenged the Ugandan Government’s failure to provide basic maternal health services as a violation of both the right to health as well as the right to life. The supreme court acknowledged that access to proper maternal health care and emergency obstetric care is fundamental to ensuring women’s constitutional rights to health and life. Such a judgment makes judicial enforcement of economic social rights such as on the right to health almost as good as having it directly provided for under a country’s constitution, and gives good basis for its justiciability and legal enforceability.

While there may be a duty to respect, protect, fulfil and even remedy the right to health, these duties will often be meaningless in practice without a clear identification of the necessary duty bearers to enforce them. The law is the starting point for this. However, the law can also be used as a double edged sword, both to facilitate as well as deter enjoyment of this right. There are legal challenges where for instance the legal profession and judiciary are inept in living up to the foresightedness and innovativeness required in applying international human rights law to expound on, and enforce state duties. Legal concepts such as the ‘Political Question’ have sometimes been applied [62]. The Political question doctrine holds that certain issues should not be decided by courts because their resolution is committed to another branch of government and that those issues are not capable, for one reason or another, of judicial resolution. Its purpose is to distinguish the role of the judiciary from those of the Legislature and the Executive, preventing the former from encroaching on either of the latter. Under this rule, courts may choose to dismiss the cases even if they have jurisdiction over them. In such instances, the courts fail to assertively use their mandate to enforce economic social rights even where they can. This was the case in *Centre for Health Human Rights & Development and 3 Ors v Attorney General* [61] where the court acknowledged that the Ugandan government had not allocated enough resources to the health sector and in particular the maternal health care services but was reluctant to determine the questions raised in the petition. It based its decision on the assertion that the court had no power to determine or enforce its jurisdiction on matters that require analysis of government health sector policies as this would be substituting its discretion for that of the executive granted to it by law.

Recognizing the right to health should generate positive health outcomes and promote the realization of health rights [63]. The duty to respect, protect and fulfil should considers each person as a moral equal although as discussed above, human rights without clear identification of duty bearers have their limitations.

## Conclusion

It is clear that while there may be a duty to respect, protect, fulfil and even remedy the right to health, these duties will often be meaningless in practice without a clear identification of the necessary duty bearers to enforce them. The law is the starting point for this to not only enshrine the right to health as a legally enforceable one but also to clearly identify duty bearers. Without this, the human right to health as outlined under international and regional human rights law generates an implausible, or even impossible, profusion of duties. Additionally, sound implementation is a necessary step to concretise the enjoyment of this right. There remains much work still to be done especially on the moral and legal fronts in order to fully guarantee the right to health. The nuances of these debates have become more obvious in the light of the current COVID 19 pandemic. The moral seeds have been planted and the legal fruit needs careful nurturing.

## Data Availability

This was a qualitative study that relied on literature review and scholarly works. Experiences of the right to health were sought from several parts of the world including countries in Africa and South Asia.
